# Sustainable non-ionic surfactants from epoxidised linseed oil: synthesis, interfacial properties, foaming behaviour, and emulsification performance

**DOI:** 10.1039/d6ra05443a

**Published:** 2026-08-26

**Authors:** Joseph Ogunjobi, Osaretin Omoruyi

**Affiliations:** a Department of Chemistry, The Federal University of Technology Akure PMB 704 Nigeria josephogunjobi@outlook.com jkogunjobi@futa.edu.ng; b DWI-Leibniz Institute for Interactive Materials, RWTH Aachen University Forckenbeckstr. 50 D-5074 Aachen Germany

## Abstract

The development of sustainable surfactants from renewable resources is essential for reducing dependence on petroleum-derived chemicals. Herein, a series of bio-based non-ionic surfactants was synthesised from epoxidised linseed oil (ELO) through heterogeneous catalyst-assisted epoxide ring-opening with poly(ethylene glycol) (PEG) and monomethyl poly(ethylene glycol) (MePEG) of varying molecular weights. Five surfactants, designated ELO400, ELO400M, ELO750M, ELO1050 and ELO1500, were successfully prepared and characterised by FT-IR, NMR and size exclusion chromatography, confirming efficient PEG grafting onto the triglyceride backbone. The surfactants exhibited excellent interfacial activity, reducing the equilibrium surface tension of water to as low as 36.64 mN m^−1^ and displaying low critical micelle concentrations (CMC) of 0.96–1.15 g L^−1^. Dynamic surface tension measurements demonstrated efficient adsorption at the air–water interface, while Gibbs adsorption analysis indicated favourable molecular packing at the interface. Foam generation increased with PEG chain length, whereas shorter PEG chains produced more persistent foams. All surfactants formed stable emulsions in a demanding 50 : 50 toluene/water system, substantially exceeding the oil loading encountered in most industrial emulsification processes. Emulsion stability increased with PEG molecular weight, with ELO1500 maintaining a partially stable emulsion for more than 50 days. Cloud point increased systematically with ethylene oxide content and decreased slightly in the presence of inorganic salts in the order Na_2_SO_4_ > NaCl > NaNO_2_, consistent with Hofmeister effects. These findings demonstrate that PEG-functionalised epoxidised linseed oil is a versatile renewable platform for producing high-performance non-ionic surfactants with tunable interfacial properties for detergency, emulsification, coatings, personal care formulations and other sustainable industrial applications.

## Abstract 2

Renewable non-ionic surfactants from epoxidised linseed oil showed low CMC and excellent surface activity. PEG chain length tuned interfacial properties, with longer chains enhancing emulsion stability and performance comparable to commercial agents.

## Graphical abstract

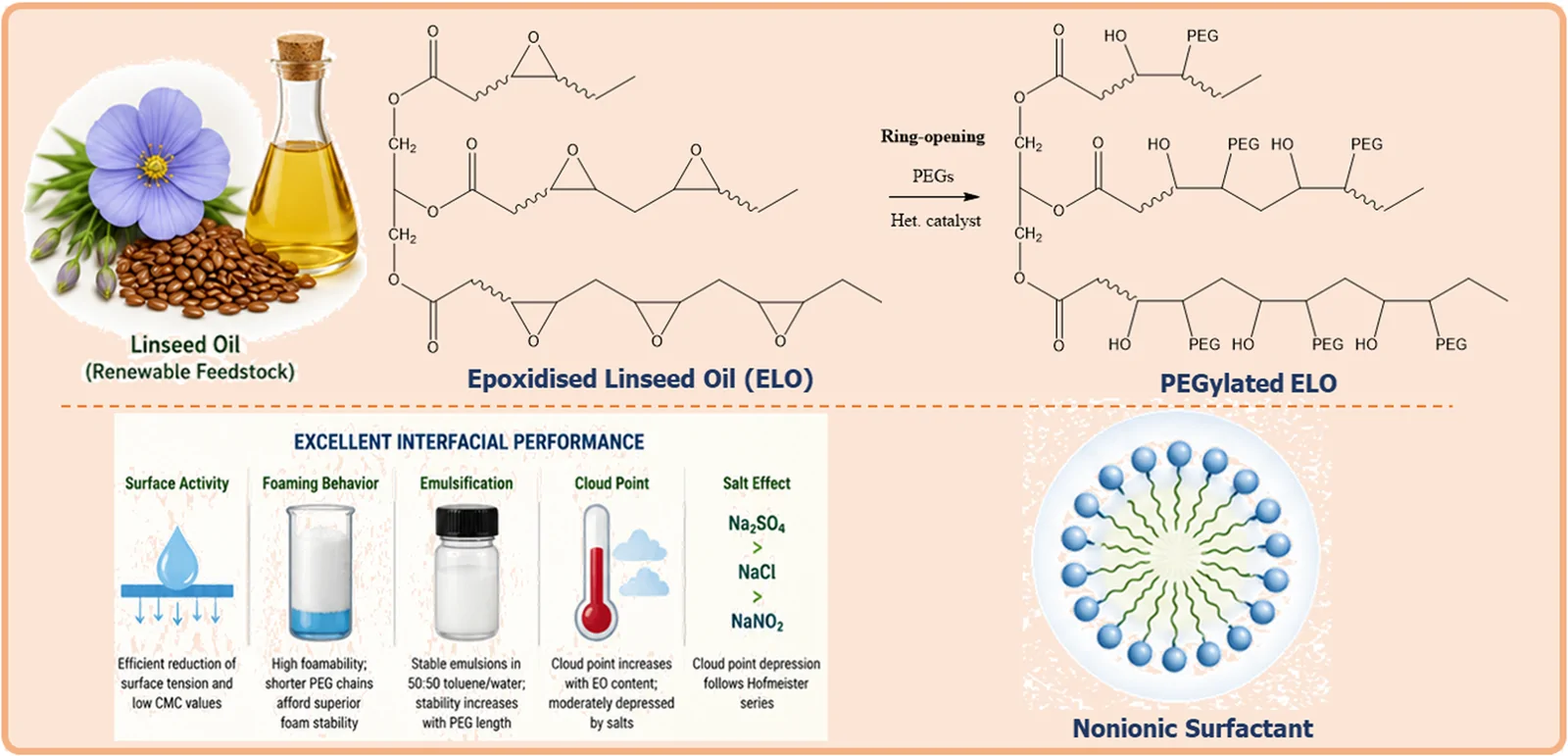

## Introduction

Linseed oil, obtained primarily from the mature seeds of the flax plant (*Linum usitatissimum*), is one of the most highly unsaturated vegetable oils and has attracted considerable interest as a renewable feedstock for industrial and chemical applications. Its fatty acid composition consists predominantly of α-linolenic acid (C18 : 3, ∼53%), together with linoleic acid (C18 : 2, ∼14%), oleic acid (C18 : 1, ∼18%), and smaller amounts of saturated fatty acids such as palmitic and stearic acids (∼9%).^1,2^ The high degree of unsaturation in linseed oil provides multiple reactive sites that can undergo chemical transformation, thereby enabling the production of value-added functional materials. Among the various modifications employed, epoxidation has emerged as one of the most important strategies for enhancing the chemical utility of vegetable oils.

Epoxidation converts carbon–carbon double bonds into oxirane rings, producing epoxidised linseed oil (ELO), a highly reactive intermediate with broad industrial relevance. ELO is regarded as an attractive bio-based platform chemical because it is renewable, biodegradable, relatively less toxic, and highly functionalized. Historically, ELO has been widely employed as a secondary plasticiser and thermal stabiliser in poly(vinyl chloride) (PVC) formulations, where the epoxide groups act as hydrogen chloride scavengers during thermal degradation.^3,4^ Nevertheless, limitations associated with its use in PVC systems, including migration, exudation, incompatibility during prolonged ageing, and surface tackiness upon exposure to heat or ultraviolet radiation, have limited its long-term use in certain formulations.

Beyond its conventional use as a plasticiser, ELO has attracted growing attention in oleochemical synthesis owing to the high reactivity of its oxirane groups. Ring-opening reactions of ELO enable the introduction of a wide range of functional groups, facilitating the synthesis of polyols, thermosetting resins, crosslinked networks, and amphiphilic derivatives. ELO-derived materials have been investigated as binders for coatings, adhesives, inks, and lubricants, as well as biobased precursors for composite materials, owing to their favourable flexibility, compatibility with polymeric matrices, and oxidative curing behaviour. Copolymerisation of ELO with epoxide-containing monomers such as cyclohexene oxide has been shown to improve the mechanical and thermal properties of the resulting epoxy systems, demonstrating the versatility of ELO as a renewable polymer precursor. Systems, demonstrating the versatility of ELO as a renewable polymer precursor.^5^

In recent years, growing environmental concerns and increasing demand for sustainable surfactants have stimulated interest in the use of vegetable oil derivatives as alternatives to petroleum-derived surfactants. Conventional surfactants are often produced from non-renewable feedstocks and may exhibit poor biodegradability and ecotoxicity. In contrast, surfactants derived from natural oils offer advantages such as renewability, structural diversity, biodegradability, and lower environmental impact. Epoxidised vegetable oils are particularly attractive intermediates because the oxirane functionality can readily undergo nucleophilic ring opening in the presence of hydrophilic molecules to generate amphiphilic structures.

The physicochemical properties of ELO-derived surfactants can be tailored through controlled functionalisation. Manipulation of the degree of epoxidation influences both the reactivity and the number of accessible functional sites on the triglyceride backbone, thereby affecting the hydrophilic–lipophilic balance (HLB) of the resulting surfactants.^6^ This tunability is important in designing surfactants for specific applications such as detergency, emulsification, dispersion, wetting, and foaming. Poly(ethylene glycol) (PEG), in particular, is widely used as a hydrophilic moiety because of its water solubility, low toxicity, flexibility, and commercial availability. Ring opening of epoxidised oils with PEG chains, therefore, provides a straightforward route toward non-ionic surfactants with potentially enhanced interfacial and self-assembly properties.

Previous studies have reported the synthesis of surfactants from epoxidised fatty compounds through epoxide ring-opening reactions.^7,8^ However, these studies have largely focused on low-molecular-weight systems, mono-functionalized derivatives, or compounds possessing relatively short hydrophobic and hydrophilic segments. Reports describing the direct functionalisation of triglyceride-based epoxidised oils with multiple PEG chains remain scarce. In particular, little attention has been devoted to the synthesis of highly functionalized PEGylated triglyceride surfactants derived directly from ELO.

In this study, a series of novel PEGylated surfactants was synthesised from ELO through catalytic epoxide ring-opening reactions with PEG and methoxy PEG (MePEG). The synthesised surfactants were comprehensively characterised using spectroscopic and chromatographic techniques. Their surface-active properties were evaluated by measurements of surface tension, critical micelle concentration (CMC), foam capacity and stability, emulsion capacity and stability, and cloud point. The work demonstrates the potential of ELO as a sustainable platform for the delivery of bio-based non-ionic surfactants with tunable physicochemical properties.

## Results and discussion

### Synthesis of ELO-based surfactants

**Fig. 1:**
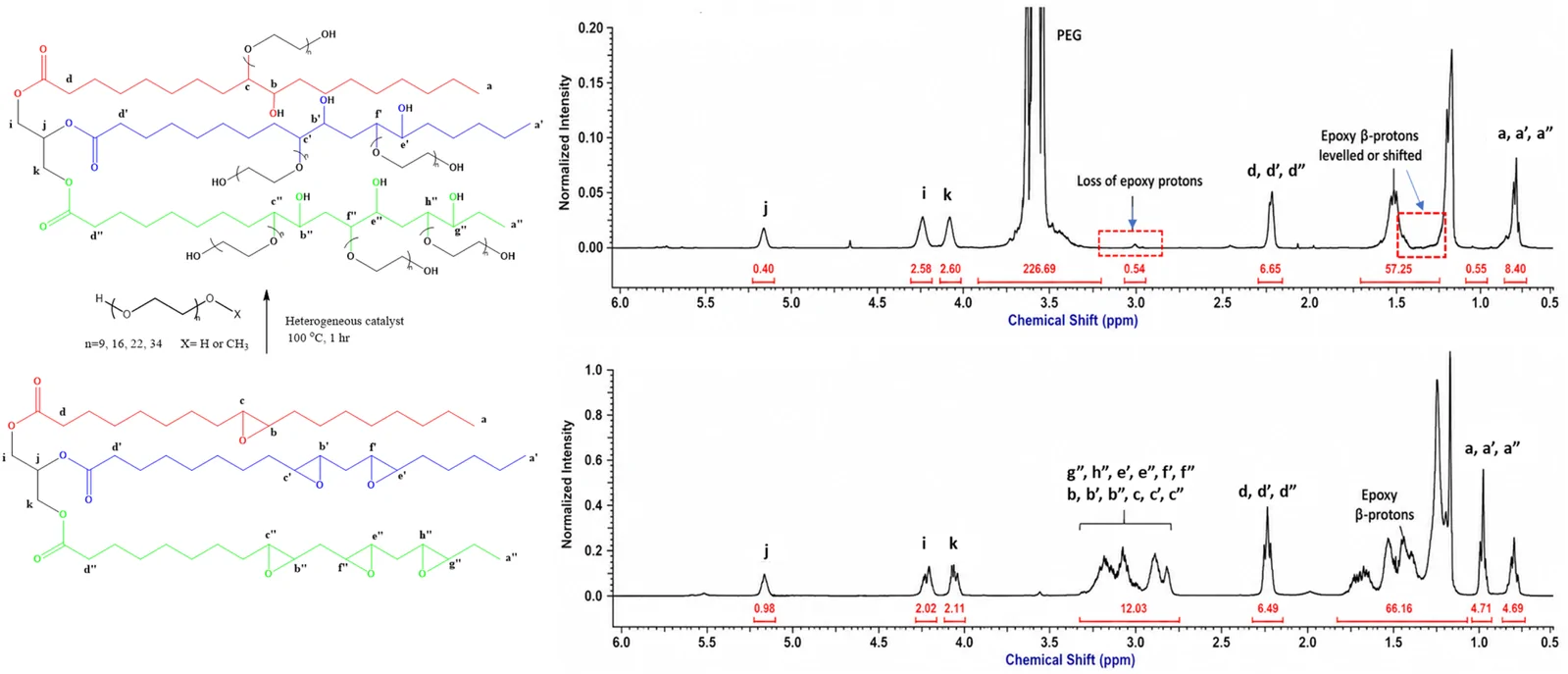
Ring-opening reaction of ELO with PEGs of varying chain length *via* a montmorillonite clay-based catalyst (left) and proton NMR spectra of ELO (bottom) and synthesised ELO400 (top) when *n* = 9.

Novel epoxidised linseed oil (ELO)-based surfactants were synthesised *via* ring-opening of the epoxide groups with PEG and MePEG, following a previously reported, greener methodology.^9^ In these reactions, a molar ratio of 6 : 1 (PEG or MePEG to epoxide functionality) was employed (Fig. 1). Due to the high viscosity of ELO, it was first diluted with toluene to facilitate handling and controlled addition. The resulting solution was then added dropwise to molten PEG or MePEG in the presence of a catalyst. The reactions yielded five PEGylated surfactants designated as ELO400, ELO1050, ELO1500, ELO400M, and ELO750M according to the type of PEG employed during the epoxide ring-opening reaction.

The numerical value in each label corresponds to the molecular weight (or PEG grade) of the PEG used in the synthesis. The suffix “M” denotes the use of monomethyl-capped PEGs in the ring-opening process. ELO400 and ELO400M were obtained as viscous dark brown liquids, whereas ELO1050, ELO1500, and ELO750M were isolated as dark brown solids. The dark brown appearance is attributed predominantly to the ring-opened epoxidised linseed oil, although trace iron species originating from the catalyst may also contribute to the observed colour. Previous ICP-MS analysis of analogous oleate-based surfactants synthesised under similar conditions confirmed the presence of minor iron leaching from the catalyst into the final products.^9^ The PEGylated surfactants were isolated in high yields ranging from 80.7 to 94.8% following catalyst removal and purification. The high recoveries indicate that the epoxide ring-opening reaction proceeded efficiently under the reaction conditions employed, with only minor losses occurring during filtration, aqueous washing and solvent removal.

^1^H NMR spectroscopy further supported the structural modification. In the spectrum of ELO (Fig. 1, bottom), epoxy proton signals (assigned to positions g″, h″, e′, e″, f′, f″, b, b′, b″, c, c′, and c″) appeared in the range of 2.5–3.5 ppm, with a total integration corresponding to 12 protons. These signals, along with the associated β-proton resonances, were absent in the spectrum of PEG-modified ELO (ELO400) (Fig. 1, top), confirming no residual epoxide detected by NMR. Additionally, the methyl resonance (peak a″) associated with the fatty acid chain bearing three epoxide groups exhibited an upfield shift following the reaction, indicating changes in the local electronic environment.

The proton and carbon signals corresponding to the carbon atoms bonded to PEG chains were assigned to 3.07–3.09 ppm in the ^1^H NMR spectrum (with an integration of approximately one proton) and 84.55 ppm in the ^13^C NMR spectrum, as corroborated by HSQC analysis. Signals associated with hydroxyl-bearing carbons overlapped with PEG methylene protons in the ^1^H NMR spectrum; however, a distinct resonance at 73.40 ppm in the ^13^C NMR spectrum was attributed to these carbons. Apart from these changes, most other resonances retained similar chemical shift positions, indicating that the triglyceride backbone remained largely intact following functionalisation. It must be noted that, there are possibilities of having the PEG chain attached to either position of the oxirane ring with the corresponding –OH group in the alternative position, thus producing multiple regioisomers.

Fourier transform infrared (FT-IR) spectroscopy was used to confirm the chemical transformation from ELO to the corresponding surfactants. Successful ring-opening and PEGylation were evidenced by the disappearance of the characteristic epoxy symmetric deformation band at 822 cm^−1^ in all products. Concurrently, the methylene C–H symmetric stretching vibrations became more pronounced with increasing PEG chain length, reflecting the growing contribution of the polyether segment. A strong absorption band corresponding to the C–O–C ether linkage was consistently observed in the region 1102–1110 cm^−1^. The broad O–H stretching band, centred at approximately 3454 cm^−1^, decreased in intensity as the PEG chain length increased, consistent with reduced relative hydroxyl content. Complete FT-IR spectral assignments for all synthesised surfactants are provided in the SI Section 1.0.

The molecular weight distributions of the synthesised PEGylated epoxidised linseed oil (ELO) surfactants were investigated by size exclusion chromatography (SEC) to provide additional evidence for successful functionalisation of the triglyceride backbone. The SEC chromatograms (Fig. S1–S4, SI Section 2.0) revealed broad molecular weight distributions for all surfactants, characteristic of products derived from naturally occurring triglycerides and statistical epoxide ring-opening reactions. The chromatograms exhibited a dominant low-molecular-weight peak together with shoulders extending towards higher molecular weights, indicating the presence of molecules with different degrees of PEG substitution and, possibly, small amounts of oligomeric species formed during synthesis.

The number-average molecular weights (*M*_n_) determined by SEC increased systematically with increasing PEG molecular weight, from 1077 Da (ELO400) to 1222 Da (ELO750M), 1901 Da (ELO1050) and 2017 Da (ELO1500) (Table S1). Similarly, the weight-average molecular weights (*M*_w_) ranged from 2697 to 6552 Da, while the dispersity indices (*Đ* = *M*_w_/*M*_n_) varied between 2.21 and 3.45, confirming that the products comprise heterogeneous molecular populations rather than discrete compounds. Such relatively broad dispersities are expected for multifunctional triglyceride-based surfactants because both the linseed oil precursor and the ring-opening reaction generate statistical distributions of substitution patterns.

To assess the SEC results, the experimentally determined molecular weights were compared with the theoretical molecular weights calculated from the average ethoxylate number. Quantitative NMR analysis confirmed that the epoxidised linseed oil contained approximately six epoxide groups per triglyceride, and the theoretical molecular weights were therefore calculated assuming complete PEG attachment at these six reactive sites. This gave theoretical molecular weights of approximately 3440 Da (ELO400), 5380 Da (ELO750M), 6870 Da (ELO1050) and 10 000 Da (ELO1500).

The SEC-derived molecular weights were substantially lower than these theoretical values. This discrepancy is not unexpected and arises principally because conventional SEC provides apparent molecular weights based on hydrodynamic volume relative to linear polymer calibration standards rather than absolute molecular masses. The synthesised surfactants possess compact, highly branched PEGylated triglyceride architectures, which occupy a smaller hydrodynamic volume than linear polymers of equivalent molecular weight and therefore elute as apparently smaller molecules. In addition, the theoretical calculations assume complete substitution of all six epoxide groups on every triglyceride molecule. In practice, the products are expected to comprise molecules with varying degrees of PEG substitution owing to the statistical nature of the ring-opening reaction and the intrinsic compositional heterogeneity of epoxidised linseed oil. Consequently, the SEC molecular weights represent average apparent values for a distribution of partially and highly substituted species rather than fully substituted molecules alone.

Despite the lower absolute molecular weights obtained by SEC, the progressive increase in *M*_n_ with increasing PEG molecular weight, together with the disappearance of the epoxide resonances in the FT-IR and NMR spectra and the appearance of new PEG-related resonances, provides strong complementary evidence for successful PEGylation of the epoxidised linseed oil. The SEC results therefore support the proposed structures while highlighting the heterogeneous nature of the synthesised surfactants.

### Dynamic surface tension, surface tension and CMC of synthesised surfactants

**Fig. 2:**
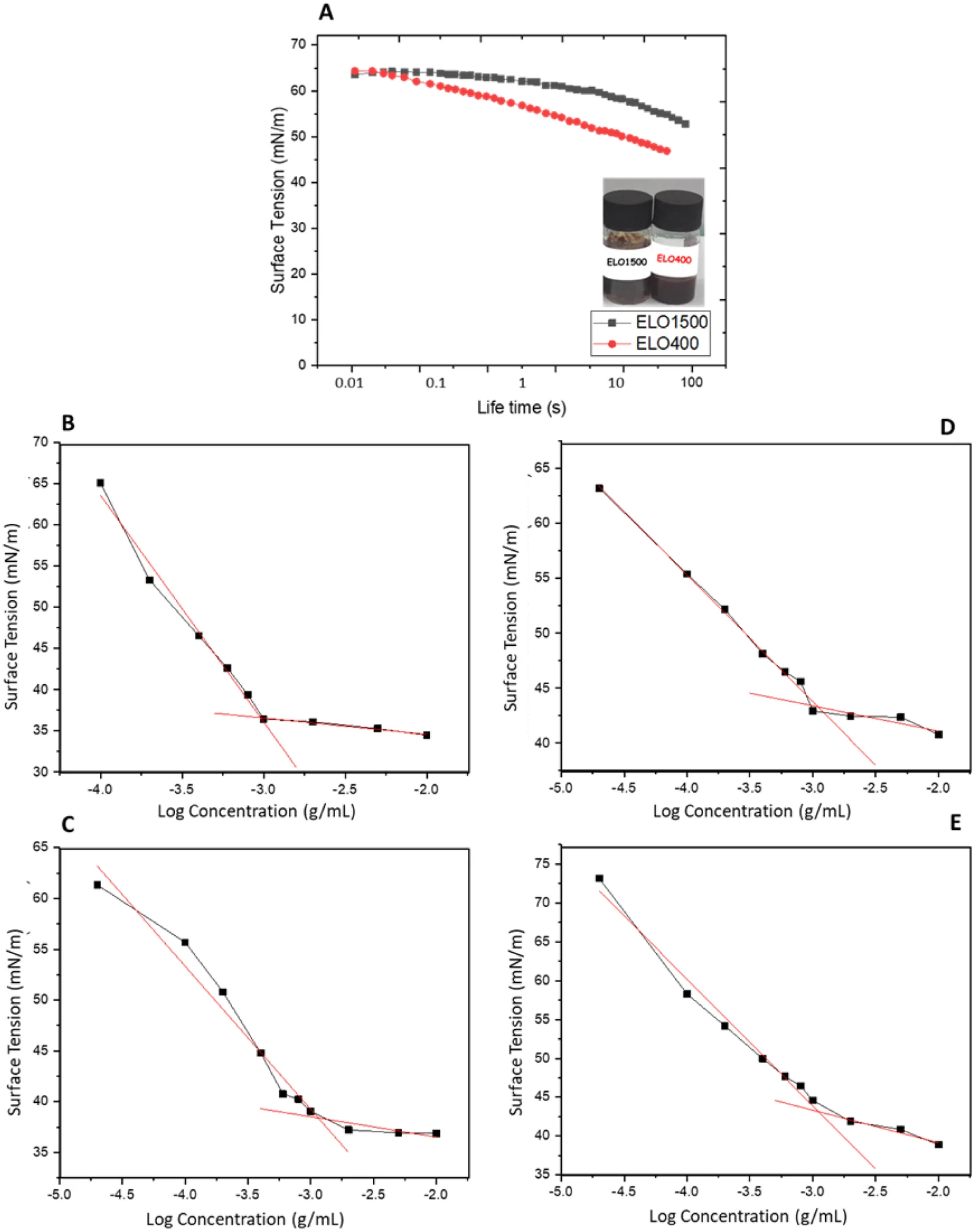
Dynamic surface tension of ELO400 and ELO1500 surfactants (A), interfacial surface tension plots for surfactants from ELO400 (B), ELO750M (C), ELO1050 (D) and ELO1500 (E). The plotted concentration is expressed in g mL^−1^, while the CMC values reported in Table 1 are expressed in g L^−1^ and mol L^−1^ after conversion using the theoretical molecular weights of the surfactants.

Dynamic surface tension refers to the variation in surface tension before reaching equilibrium at the interface. This is particularly relevant in detergent formulations, where equilibration may occur over extended timescales. In this study, dynamic surface tension was measured using a maximum bubble pressure tensiometer, enabling evaluation of the time-dependent surface activity of the synthesised surfactants over a period of up to 100 s. Representative results for ELO400, containing the shortest PEG chain, and ELO1500, containing the longest PEG chain, are presented in Fig. 2(A).

Both surfactants exhibited significant surface activity and favourable adsorption dynamics. ELO400 reduced the surface tension to approximately 47 mN m^−1^ within 80 s, whereas ELO1500 achieved a surface tension of about 52 mN m^−1^ after 100 s. In both cases, equilibrium had not yet been attained within the experimental timeframe, indicating relatively slow interfacial adsorption behaviour characteristic of polymeric surfactants.

Dynamic surface tension is governed by two principal processes: diffusion of surfactant molecules from the bulk solution to the interface and subsequent adsorption and rearrangement at the surface. For polymeric surfactants containing PEG hydrophilic segments, adsorption at the interface is generally regarded as the rate-determining step.^10^ As adsorption proceeds and interfacial coverage increases, the surface free energy decreases, resulting in progressive reduction of the surface tension.

The synthesised ELO-based surfactants adsorbed effectively at the air–water interface and reduced surface tension at relatively low concentrations. Equilibrium surface tension values ranged from 36.64 to 43.11 mN m^−1^, with ELO400 producing the greatest reduction in surface tension as shown in Fig. 2(B–E) and Table 1. In general, the extent of surface tension reduction decreased with increasing PEG chain length, indicating that enlargement of the hydrophilic segment diminished the ability of the surfactant molecules to pack efficiently at the interface. This behaviour is typical of non-ionic polymeric surfactants, where excessive hydrophilicity can reduce interfacial adsorption efficiency and increase steric hindrance between neighbouring molecules.^11,12^

**Table 1:** Critical micelle concentration (CMC), surface tension (γ_CMC) and effectiveness (π_CMC) of the ELO surfactants based on PEGs 400, 750, 1050 and 1500 chain length

Surfactant code	*γ*_CMC_ (mN m^−1^)	CMC (g L^−1^)	CMC (mol L^−1^) ×10^−4^	*Γ*_max_ (×10^−6^ mol m^−2^)	*A*_min_ (nm^2^ per molecule)	π_CMC_ (mN m^−1^)
ELO400	36.64	0.96	2.78	4.93	0.34	36.16
ELO750M	38.46	1.15	2.14	4.23	0.39	34.34
ELO1050	43.29	1.08	1.58	3.64	0.46	29.51
ELO1500	43.11	1.12	1.11	3.90	0.43	29.69

The surfactants exhibited low critical micelle concentrations (CMCs), ranging from 1.0 to 1.2 g L^−1^ (equivalent to 1.1–2.8 × 10^−4^ mol L^−1^; Table 1), indicating a strong propensity for micelle formation. Quantitative ^1^H NMR analysis established an average of six epoxide groups per triglyceride in the epoxidised linseed oil. Accordingly, the theoretical molecular weights, derived from the average degree of epoxidation and PEG incorporation, were used to calculate the molar surfactant concentrations and the corresponding CMC values expressed in mol L^−1^, rather than the apparent molecular weights obtained by SEC, which reflect hydrodynamic volume rather than absolute molecular mass. The relatively low CMC values suggest that only small amounts of surfactant were required to saturate the interface and initiate self-assembly into micellar aggregates. Such behaviour is advantageous in industrial formulations because lower surfactant dosages are needed to achieve desired interfacial properties, thereby reducing formulation cost and minimising residual surfactant loading in aqueous systems.^13^

The CMCs expressed in mass concentration (g L^−1^) provide a clearer indication of the influence of PEG chain length on the aggregation behaviour of the synthesised surfactants. ELO400 exhibited the lowest CMC (1.0 g L^−1^), indicating that the surfactant required the smallest mass concentration to initiate micellisation. As the PEG chain length increased, the CMC increased slightly to 1.1 g L^−1^ for ELO1050 and for ELO1500, while the methyl-capped analogue ELO750M exhibited the highest CMC (1.2 g L^−1^). This trend is consistent with the behaviour commonly reported for conventional ethoxylated non-ionic surfactants, where increasing the number of ethylene oxide units enhances the hydrophilicity and hydration of the surfactant molecules, thereby increasing their solubility in water and reducing the thermodynamic driving force for micellisation.^11^ Consequently, higher concentrations are required before aggregation becomes favourable.^12^

The difference between the trends observed for molar and mass-based CMC arises primarily from the substantial increase in molecular weight accompanying PEG chain extension. Although the molar CMC decreases because fewer molecules are required to form micelles, each molecule contributes a considerably greater mass. Consequently, when expressed in g L^−1^, the CMC increases with PEG molecular weight, reflecting the greater amount of surfactant required on a mass basis to reach the critical aggregation concentration.

The relatively small variation in mass-based CMC (1.0–1.2 g L^−1^) further suggests that all the synthesised surfactants possess strong surface activity despite differences in PEG chain length. Their comparable CMC values indicate that the multifunctional triglyceride backbone provides an effective hydrophobic domain, while variation in PEG length allows tuning of interfacial properties without substantially compromising the molecules' ability to self-assemble in aqueous solution.

The sharp decline in surface tension observed near the CMC region further confirms efficient interfacial adsorption and cooperative aggregation behaviour.

The Gibbs adsorption parameters provide further insight into the interfacial packing behaviour of the PEGylated linseed oil surfactants. Maximum surface excess values were of the order of 3.6–4.9 × 10^−6^ mol m^−2^, which are typical of efficient non-ionic surfactants possessing large hydrophobic groups. Correspondingly, the minimum molecular area ranged from 0.34 to 0.46 nm^2^ per molecule. Among the series, ELO400 exhibited the highest surface excess and the smallest molecular area, indicating the most compact adsorption at the air/water interface. This observation agrees with its lowest equilibrium surface tension and reflects the relatively short PEG chains, which minimise steric hindrance and permit closer molecular packing. As PEG molecular weight increased, the surface excess decreased slightly while the molecular area increased, consistent with the progressively larger hydrophilic head groups occupying greater interfacial area. The increase in steric crowding reduces the number of molecules that can adsorb per unit surface area, despite the improved aqueous solubility of the surfactants. Interestingly, ELO750M exhibited a slightly greater surface excess than ELO1050 despite containing methyl-capped PEG chains. The absence of a terminal hydroxyl group reduces hydration of the PEG segment, allowing somewhat closer interfacial packing than the corresponding hydroxyl-terminated analogue. Nevertheless, the effect is modest because adsorption is governed primarily by the balance between the bulky triglyceride core and the multiple PEG substituents.

The effectiveness of the surfactants, expressed as the difference between the surface tension of pure water and the surface tension at the CMC, ranged from approximately 29 to 36 mN m^−1^ (Table 1). These values compare favourably with several commercial non-ionic surfactants, including ethoxylated fatty alcohols, sorbitan esters (Tweens), and alkyl polyglucosides, which commonly exhibit equilibrium surface tensions between 35 and 45 mN m^−1^ and CMC values in the order of 10^−4^ to 10^−5^ mol L^−1^.^12,13^ The ELO-derived surfactants therefore demonstrate competitive interfacial performance while offering the additional advantages associated with renewable triglyceride feedstocks, including biodegradability, lower toxicity, and reduced dependence on petroleum-derived raw materials.^14,15^

The observed behaviour also highlights the importance of hydrophilic–lipophilic balance (HLB) in determining surfactant performance. Surfactants with shorter PEG chains possessed stronger hydrophobic character, favouring adsorption and tighter molecular packing at the interface, whereas longer PEG chains improved aqueous solubility but reduced interfacial activity. The monomethyl-capped derivatives additionally showed slightly altered surface behaviour, likely due to reduced hydrogen bonding and changes in molecular hydration compared with hydroxyl-terminated PEG analogues.^16^

The low CMC values and substantial reductions in surface tension observed demonstrate that PEGylated ELO derivatives are effective bio-based non-ionic surfactants with potential applications in detergency, emulsification, coatings, cosmetics, and enhanced oil recovery systems.

ELO400M was not included in selected analyses because the available sample was exhausted during the course of the study and insufficient material remained for repeat preparation and subsequent characterisation.

### Foam capacity and stability

**Fig. 3:**
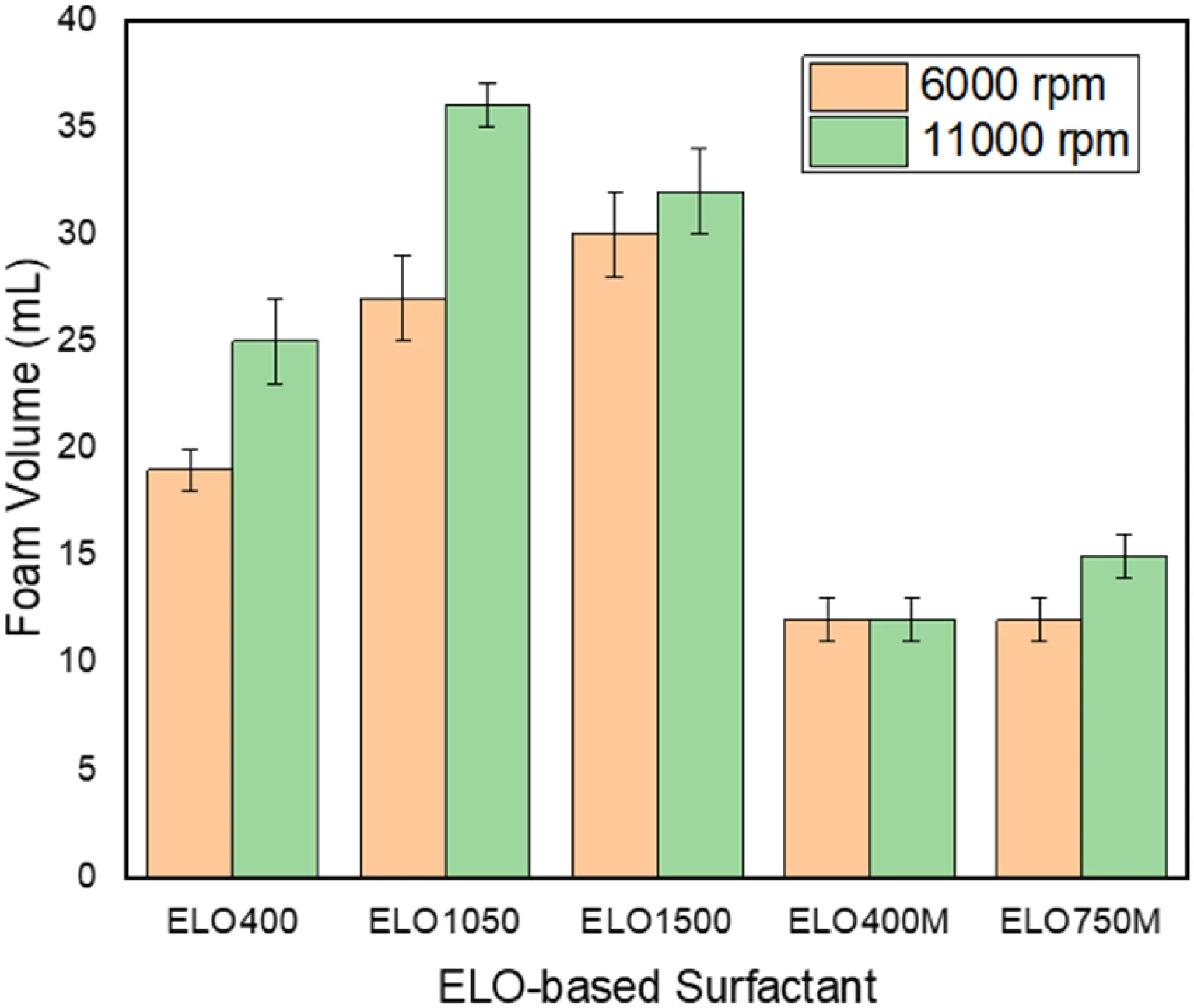
Foam capacity of 0.25% surfactant solution from ELO400, ELO1050 & ELO1500 and ELO400M and ELO750M; and the influence of stirring speed on foam volume. Stirring speeds: 6000 rpm and 11 000 rpm.

Foam capacity is an important performance parameter for surfactants intended for applications such as detergency, cosmetics, personal care products, and enhanced oil recovery. The foaming behaviour of the synthesised ELO-based surfactants was evaluated at two homogenisation speeds, 6000 and 11 000 rpm, and the resulting foam volumes are presented in Fig. 3. The results demonstrate that both surfactant structure and agitation intensity significantly influenced foam formation.

Among the uncapped PEGylated surfactants, foam formation generally increased with increasing ethylene oxide content. At 6000 rpm, foam volumes increased from approximately 19 mL for ELO400 to 27 mL for ELO1050 and reached a maximum of about 30 mL for ELO1500. A similar trend was observed at 11 000 rpm, where ELO400, ELO1050, and ELO1500 generated approximately 25, 36, and 32 mL of foam, respectively. The increased foamability with longer PEG chains can be attributed to enhanced hydrophilicity and improved stabilisation of the air–water interface by the larger polyether segments. Increased ethylene oxide content generally improves water solubility and promotes the formation of more elastic interfacial films, which favour foam generation and stabilisation.^12^

The monomethyl-capped surfactants, ELO400M and ELO750M, exhibited significantly lower foam capacities compared with their hydroxyl-terminated analogues. ELO400M generated approximately 12 mL of foam at both stirring speeds, indicating little improvement in foam generation despite increased agitation intensity. Similarly, ELO750M produced only modest foam volumes of approximately 12 and 15 mL at 6000 and 11 000 rpm, respectively. The reduced foaming behaviour of the methyl-capped surfactants is attributed to the replacement of the terminal hydroxyl group with a methyl group, thereby reducing overall hydrophilicity and hydrogen-bonding capability. The hydroxyl end group in uncapped PEG surfactants contributes significantly to intermolecular interactions with water and facilitates the formation of more stable interfacial films.^17^ Consequently, methyl termination reduces the ability of the surfactant molecules to stabilise entrapped air bubbles during homogenisation.

The influence of stirring speed on foam generation was also evident. In general, increasing the homogenisation speed from 6000 to 11 000 rpm increased foam volume for most surfactants. Higher agitation speeds introduce more air into the system and increase bubble generation frequency, resulting in greater foam formation. ELO400 and ELO1050 showed the most pronounced increase in foam volume with increasing stirring speed, while ELO1500 displayed only a slight increase. The smaller increase observed for ELO1500 may be related to its higher molecular weight and increased solution viscosity, which can hinder rapid diffusion and rearrangement of surfactant molecules at newly created interfaces during intense agitation. Polymeric surfactants containing longer PEG chains are known to exhibit slower adsorption kinetics due to steric effects and reduced mobility in solution.^18^

Interestingly, ELO400M exhibited essentially identical foam volumes at both stirring speeds, suggesting that agitation alone could not compensate for the poorer interfacial properties associated with the methyl-capped structure. This further supports the importance of terminal hydroxyl functionality in promoting foamability in these triglyceride-derived surfactants.

Comparison with conventional commercial surfactants further highlights the promising foaming characteristics of the ELO-based materials. Reported foam capacities for cetyltrimethylammonium bromide (CTAB), sodium dodecyl sulfate (SDS), and Tween 80 under similar experimental conditions are generally lower or comparable, particularly when considering the relatively low concentrations employed for the ELO surfactants.^19^ Although SDS is typically regarded as a highly foaming surfactant, the ELO400, ELO1050, and ELO1500 systems exhibited competitive or superior foam generation, especially at lower stirring speeds. Tween 80, a commercial non-ionic surfactant, is generally characterised by relatively low foam production due to its large hydrophilic head group and bulky structure. The superior foam capacity of the uncapped ELO-based surfactants, therefore, indicates that the balance between the hydrophobic triglyceride backbone and PEG hydrophilic chains was favourable for foam generation.

The results demonstrate that both PEG chain length and terminal functionality strongly influence the foaming properties of the synthesised surfactants. Increasing PEG chain length enhanced foam formation up to an optimum level, while methyl capping reduced foamability due to decreased hydrophilicity. Overall, the surfactants, particularly ELO1050 and ELO1500, exhibited promising foam-generating properties comparable with or superior to several commercial surfactants, supporting their potential application in detergents, emulsifiers, and personal care formulations.

**Fig. 4:**
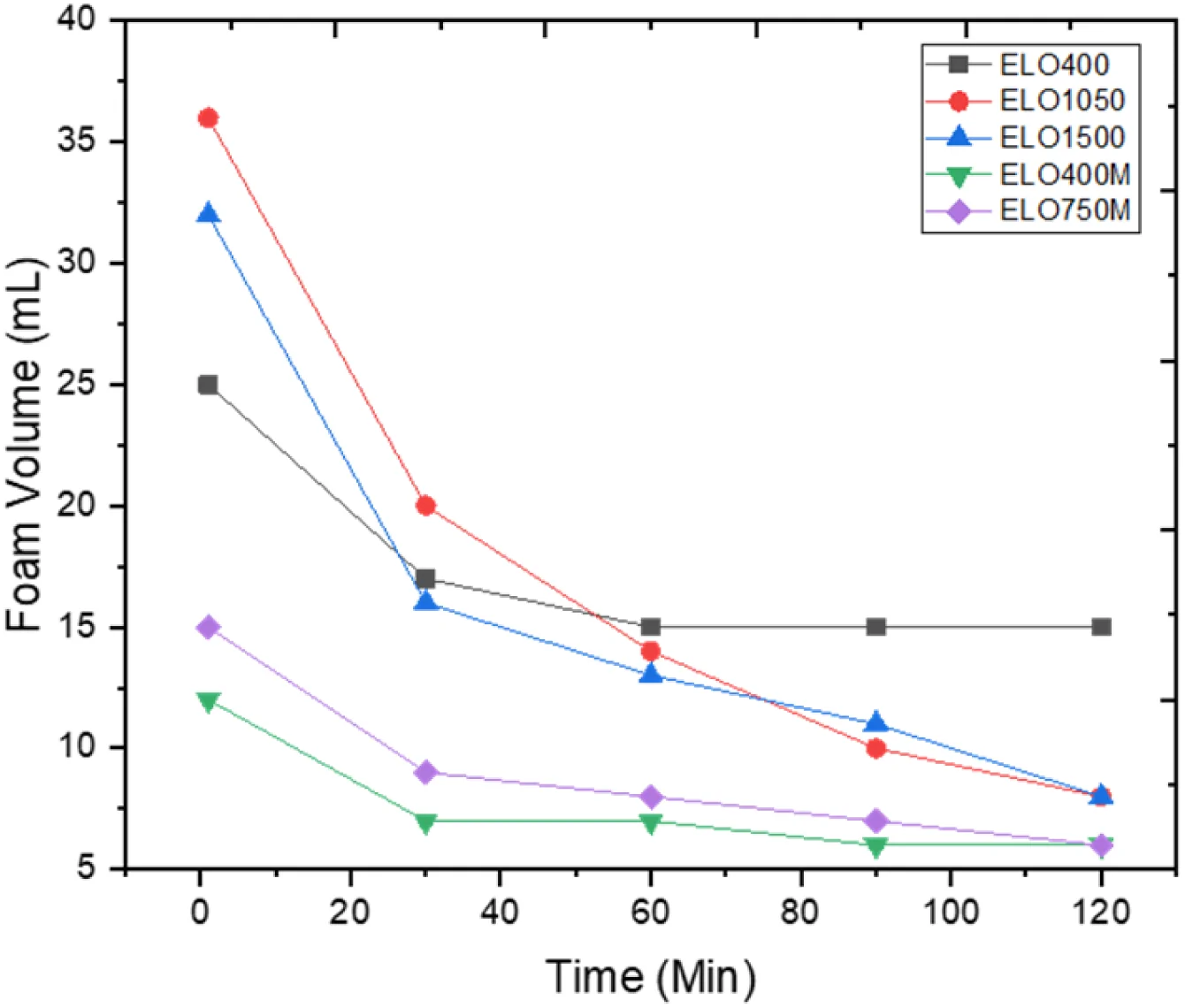
Foam stability of epoxidised linseed oil-based surfactants from ELO400, ELO1050, ELO1500, ELO400M and ELO750M (0.25% surfactant concentration).

The foam stability of the synthesised linseed oil-based surfactants was evaluated by monitoring the decay in foam volume over 120 min following foam generation at 11 000 rpm (Fig. 4 and Table S2 in the SI Section 3.0). Foam retention was calculated relative to the initial foam volume measured after 1 min, while foam reduction was expressed as the percentage decrease from the initial volume. The results demonstrate distinct differences in foam persistence among the surfactants, reflecting the influence of PEG chain length and terminal functionality on foam stability.

Within the first 30 min, all surfactants exhibited a marked decrease in foam volume, indicating rapid initial drainage of the liquid films separating adjacent bubbles. ELO400 retained 68.0% of its initial foam volume (17 mL from 25 mL), corresponding to a 32.0% reduction. ELO1050 showed a lower retention of 55.6% (20 mL from 36 mL), while ELO1500 retained 50.0% (16 mL from 32 mL). Among the methyl-capped surfactants, ELO400M retained 58.3% (7 mL from 12 mL) and ELO750M retained 60.0% (9 mL from 15 mL), corresponding to reductions of 41.7% and 40.0%, respectively. Thus, approximately 40–50% of the foam generated by most surfactants collapsed during the first 30 min, suggesting that liquid drainage was the dominant destabilisation mechanism immediately after foam formation.

After 60 min, differences in foam stability became more apparent. ELO400 retained 60.0% of its original foam volume (15 mL), representing a total reduction of 40.0%, and remained unchanged thereafter. In contrast, ELO1050 retained only 38.9% (14 mL), while ELO1500 retained 40.6% (13 mL). The methyl-capped surfactants exhibited intermediate behaviour, with ELO400M maintaining 58.3% (7 mL), identical to its 30 min value, whereas ELO750M retained 53.3% (8 mL). These observations indicate that the initial rapid drainage was followed by a slower stage of foam decay, particularly for ELO400 and ELO400M.

At 90 min, ELO400 continued to exhibit excellent stability, maintaining 60.0% of its initial foam volume (15 mL) without further collapse. Foam retention decreased to 27.8% (10 mL) for ELO1050 and 34.4% (11 mL) for ELO1500, corresponding to reductions of 72.2% and 65.6%, respectively. ELO400M retained 50.0% (6 mL), while ELO750M retained 46.7% (7 mL), indicating moderate but sustained foam stability.

After 120 min, ELO400 remained the most stable surfactant, still retaining 60.0% of its initial foam volume (15 mL). ELO400M also exhibited good stability, retaining 50.0% (6 mL). In comparison, ELO1050 and ELO1500 retained only 22.2% (8 mL) and 25.0% (8 mL) of their original foam volumes, respectively, while ELO750M retained 40.0% (6 mL). The stability ranking after 2 h was therefore:ELO400 > ELO400M > ELO750M > ELO1500 > ELO1050.

The superior foam stability of ELO400 and ELO400M is consistent with their relatively shorter PEG chains, which confer a more favourable hydrophilic–lipophilic balance for the formation of robust interfacial films. Their greater hydrophobic character likely promotes closer molecular packing at the air–water interface, producing stronger and more elastic adsorption layers that resist liquid drainage and bubble coalescence. Such densely packed interfacial films have been associated with enhanced foam stability in non-ionic surfactants and can be interpreted in terms of a more favourable critical packing parameter (CPP), which favours lamellar or closely packed interfacial structures capable of retarding film thinning.^16^ Surfactants with shorter PEG chains possess relatively larger hydrophobic contributions and lower effective head-group area, favouring closer packing of the hydrophobic chains at the interface. Such packing behaviour promotes the formation of more ordered lamellar or bilayer interfacial structures that resist liquid drainage within the foam films.^20^ These compact interfacial films increase film elasticity and mechanical strength, thereby delaying foam rupture and bubble coalescence.

Conversely, increasing PEG chain length enhanced foam generation but reduced foam persistence. The more highly hydrated PEG chains in ELO1050 and ELO1500 produced larger initial foam volumes but also formed more mobile interfacial films that were less resistant to drainage and bubble coalescence. This observation highlights the common trade-off between foaming capacity and foam stability in non-ionic surfactants, where greater hydrophilicity often promotes rapid foam formation but compromises long-term foam persistence.

Polymeric surfactants containing long ethylene oxide segments are known to exhibit slower interfacial rearrangement and weaker intermolecular cohesion within foam lamellae, contributing to progressive foam collapse over time.^18^

Overall, the results demonstrate that although all the synthesised surfactants generated stable foams over the two-hour observation period, ELO400 and ELO400M exhibited the greatest foam stability, retaining 60% and 50%, respectively, of their original foam volumes after 120 min. These characteristics suggest that these surfactants are particularly promising for applications requiring persistent foams, such as detergents, firefighting foams, flotation processes, and personal care formulations. In contrast, the higher PEG molecular weight surfactants may be better suited to applications where rapid foam generation rather than prolonged foam stability is the primary requirement.

The reduction in foam volume observed throughout the experiment can be attributed to the fundamental mechanisms responsible for foam destabilisation, namely liquid drainage, bubble coalescence, and disproportionation (coarsening).^21^ As liquid drains from the thin films separating neighbouring bubbles, the lamellae become increasingly fragile and susceptible to rupture. Gas diffusion from smaller to larger bubbles also contributes to foam instability by promoting bubble growth and eventual collapse. Surfactants capable of forming strong, elastic, and closely packed interfacial layers are therefore more effective at delaying these destabilisation processes.

Comparison with commercial surfactants demonstrates the promising performance of the ELO-derived systems. The foam stability of ELO400 and ELO400M at 0.25% concentration exceeded that reported for conventional synthetic surfactants such as cetyltrimethylammonium bromide (CTAB), sodium dodecyl sulfate (SDS), and Tween 80 under similar conditions.^19^ SDS, although known for generating high foam volumes, typically exhibits relatively rapid foam collapse due to fast liquid drainage and thinner interfacial films. Tween 80, a widely used non-ionic surfactant, generally produces lower and less stable foams because of its bulky hydrophilic structure. The superior stability observed for ELO400 and ELO400M, therefore, highlights the potential of these renewable triglyceride-derived surfactants as effective foam stabilisers for detergent, cosmetic, and industrial applications.

Overall, the results demonstrate that foam stability in the ELO-based surfactants was strongly dependent on PEG chain length and terminal functionality. Shorter PEG chains favoured stronger interfacial packing and greater resistance to foam collapse, while longer PEG chains promoted foam generation but reduced long-term stability. The excellent persistence exhibited by ELO400 and ELO400M suggests that these surfactants may serve as sustainable alternatives to conventional petroleum-derived foam stabilisers.

### Relative emulsion capacity and stability

The relative emulsion capacity and long-term emulsion stability of the synthesised ELO-based surfactants were evaluated by homogenising a 50 : 50 toluene/water mixture in the presence of 100 mg of surfactant, followed by visual observation of phase behaviour over several weeks (Fig. S5, SI Section 5.0). The emulsions were classified qualitatively according to the extent of phase separation and persistence of the emulsified layer during storage. It is important to note that the oil-to-water ratio employed in this study was considerably higher than that commonly encountered in most technical emulsions and commercial formulations. The 50 : 50 toluene/water system represents a challenging model emulsion designed to compare relative surfactant performance rather than simulate a specific commercial formulation. The ability of the surfactants to stabilise such highly concentrated biphasic systems, therefore, demonstrates exceptionally strong emulsifying performance under demanding conditions.

All surfactants were capable of initially forming emulsions, confirming that the PEGylated linseed oil derivatives possessed sufficient amphiphilic character to adsorb effectively at the oil–water interface. However, clear differences were observed in the long-term stability of the emulsions depending on PEG chain length and terminal group functionality. Based on visual stability over time, the relative emulsion stability followed the order: ELO1500 > ELO1050 > ELO750M > ELO400 > ELO400M.

Among the surfactants investigated, ELO1500 exhibited the highest emulsion stability. Although gradual phase separation occurred over time, the emulsion still appeared partially intact after 52 days, indicating remarkable long-term resistance to coalescence and separation. ELO1050 also showed excellent stability, maintaining a substantial emulsified phase for over 50 days before significant separation became evident. In contrast, ELO750M and ELO400 retained stable emulsions for shorter periods, with ELO750M remaining visibly stable for approximately 21 days. ELO400M exhibited the poorest stability, undergoing relatively rapid separation within the first week of storage.

The enhanced relative emulsion stability observed for ELO1500 and ELO1050 is attributed primarily to their higher PEG content. Longer PEG chains provide increased steric stabilisation at the oil–water interface by forming hydrated interfacial layers around dispersed droplets. These hydrated polyether chains create repulsive steric barriers that inhibit droplet aggregation and coalescence, thereby improving emulsion persistence.^22^ Non-ionic surfactants containing longer ethylene oxide segments are well known to stabilise emulsions through steric mechanisms rather than electrostatic repulsion, making them particularly effective in systems containing high oil fractions.^16^

Visual observations further suggested that the emulsions gradually separated into distinct oil and aqueous phases over time, with surfactants containing longer PEG chains forming cream-like layers at the upper region of the sample while clearer aqueous layers developed at the bottom. This behaviour is characteristic of creaming phenomena, where dispersed oil droplets migrate upward due to density differences between the dispersed and continuous phases.^23^ The persistence of the cream layer in ELO1500 and ELO1050 indicates that although macroscopic phase separation occurred gradually, significant portions of the dispersed droplets remained kinetically stabilised for extended periods.

The lower stability observed for ELO400 and especially ELO400M likely resulted from insufficient steric stabilisation arising from their shorter PEG chains. Although shorter PEG segments may improve interfacial adsorption efficiency due to increased hydrophobicity, the resulting interfacial films are generally thinner and less hydrated, making them more susceptible to droplet coalescence and eventual phase separation.^12^ The reduced stability of ELO400M further highlights the importance of terminal hydroxyl functionality in promoting interfacial hydration and stabilisation. Replacement of the hydroxyl group with a methyl group decreases hydrophilicity and hydrogen-bonding interactions with water, thereby reducing the ability of the surfactant to maintain stable dispersed droplets over prolonged periods.

The observed long-term emulsion behaviour demonstrates that PEG chain length plays a critical role in balancing interfacial adsorption and steric stabilisation. While shorter PEG chains favoured stronger surface activity and foam stability, longer PEG chains significantly enhanced emulsion persistence. The ability of ELO1500 and ELO1050 to maintain partially stable emulsions for over 50 days under such high oil loading conditions compares favourably with many commercial non-ionic surfactants used in coatings, agrochemical formulations, cosmetics, and petroleum emulsions.^24^

To this end, the results demonstrate that the synthesised ELO-based surfactants possess excellent emulsifying properties, particularly considering the highly concentrated 50 : 50 oil/water system employed. The exceptional long-term stability exhibited by ELO1500 and ELO1050 highlights the strong potential of PEGylated epoxidised linseed oil derivatives as sustainable bio-based emulsifiers for industrial applications requiring prolonged emulsion stability.

### Cloud point

Cloud point is an important characteristic of non-ionic surfactants and corresponds to the temperature at which an aqueous surfactant solution becomes turbid due to phase separation. At the cloud point, dehydration of the poly(ethylene oxide) (PEO) chains occurs, reducing surfactant solubility and leading to the formation of a surfactant-rich phase and a dilute aqueous phase.^16^ Consequently, cloud point provides a useful measure of the hydrophilic character and temperature tolerance of non-ionic surfactants.

The cloud points of the ELO-based surfactants were determined in water and in the presence of sodium sulfate, sodium chloride, and sodium nitrite. As shown in Table 2, the cloud point increased with increasing PEG chain length for both the hydroxyl-terminated and mono methyl-capped surfactants. This trend is expected because longer PEG chains contain a greater number of ethylene oxide units capable of hydrogen bonding with water, thereby increasing surfactant hydration and delaying phase separation upon heating.^12^ Thus, ELO1500 exhibited the highest cloud point, whereas ELO400 displayed the lowest among the hydroxyl-terminated surfactants.

**Table 2:** Cloud point and influence of different inorganic sodium salts (1 wt%) on the cloud point behaviours of (1 wt%) linseed oil-based surfactants. Values are means ± standard deviation of triplicate determinations. Means with different superscript in the same column are significantly different (*P* ≤ 0.5)

Surfactants	Without salt (°C)	Na_2_SO_4_ (°C)	NaCl (°C)	NaNO_2_ (°C)
ELO400	48.00^a^ ± 2.00	40.00^b^ ± 1.00	42.00^a^ ± 1.00	44.00^a^ ± 1.00
ELOM400	46.00^a^ ± 1.00	38.00^a^ ± 1.00	39.00^a^ ± 1.00	41.00^a^ ± 2.00
ELOM750	73.00^b^ ± 1.00	70.00^c^ ± 1.00	70.00^c^ ± 2.00	73.00^c^ ± 1.00
ELO1000	90.00^c^ ± 1.00	85.00^d^ ± 1.00	85.00^d^ ± 2.00	85.00^d^ ± 2.00
ELO1500	96.00^d^ ± 1.00	90.00^e^ ± 1.00	91.00^e^ ± 2.00	92.00^e^ ± 2.00

The observed relationship between cloud point and PEG content is consistent with previous reports on ethoxylated non-ionic surfactants, where increasing the number of ethylene oxide units leads to a progressive increase in cloud point due to enhanced water solubility. The results, therefore, confirm that the hydrophilic contribution of the PEG chains remains the dominant factor controlling the thermal behaviour of the synthesised surfactants.

The addition of inorganic sodium salts resulted in a reduction in cloud point for almost all surfactants studied. The magnitude of the decrease depended on the nature of the anion and generally followed the order: Na_2_SO_4_ > NaCl > NaNO_2_.

This trend is consistent with the Hofmeister series and reflects differences in the ability of the ions to compete with the ethylene oxide groups for water molecules. Sulfate ions are strongly hydrated and effectively remove water from the hydration shell surrounding the PEG chains, resulting in a pronounced salting-out effect and a corresponding decrease in cloud point. Chloride ions produced a moderate reduction, whereas nitrite ions had the least effect. In the case of ELO750M, sodium nitrite produced little or no measurable depression of the cloud point, indicating that the hydration of the surfactant remained largely unaffected under the experimental conditions.

The reduction in cloud point in the presence of salts is commonly attributed to dehydration of the polyether chains. Strongly hydrated ions reduce the availability of free water molecules for hydrogen bonding with the ethylene oxide units, decreasing surfactant solubility and promoting phase separation at lower temperatures.^25^ This explanation agrees with numerous studies on ethoxylated surfactants, including Tween 80 and Triton X-100, where sodium sulfate consistently produces a greater cloud point depression than sodium chloride.^26^

Although all three salts lowered the cloud points, the extent of the reduction was relatively small in comparison with the original cloud point values. This observation suggests that the ELO-based surfactants retain substantial hydration even in saline environments. Such behaviour is advantageous for practical applications because it indicates good tolerance toward dissolved electrolytes commonly encountered in detergent formulations, industrial cleaning systems, and enhanced oil recovery operations.

An alternative explanation proposed in recent studies is that ion-specific effects arise not only from modifications of the water structure but also from direct ion–surfactant interactions.^27^ Such interactions may influence the hydration state of the PEG chains and contribute to the observed differences among sulfate, chloride, and nitrite ions.

The cloud point results demonstrate that the ELO-based surfactants possess relatively high thermal stability in aqueous solution, particularly those containing longer PEG chains. The ability to maintain high cloud points while exhibiting only moderate sensitivity to added electrolytes compares favourably with many commercial ethoxylated non-ionic surfactants. For example, Mushtaq *et al.* reported cloud points of approximately 16, 43, and 64 °C for ethoxylated surfactants containing 7, 11, and 16 ethylene oxide units, respectively.^25^ The ELO-derived surfactants exhibited substantially higher cloud points at comparable or greater ethoxylation levels, reflecting the influence of the triglyceride-based hydrophobic backbone and the presence of multiple PEG chains per molecule. These characteristics suggest that the synthesised surfactants may be particularly suitable for applications requiring non-ionic surfactants with high cloud points and good electrolyte tolerance.

The statistical analysis confirms that addition of inorganic salts generally reduced the cloud point of the synthesized non-ionic surfactants. For ELO400, ELO400M, ELO1050, and ELO1500, significant differences were observed between the salt-free solution and one or more salt-containing systems (one-way ANOVA, *p* < 0.05). However, differences among the three salts were generally not significant, except for ELO400 where Na_2_SO_4_ produced a significantly greater reduction in cloud point than NaNO_2_. For ELO750M, although the overall ANOVA indicated treatment effects, Tukey's multiple comparison test did not identify any statistically significant pairwise differences, suggesting that the three salts had comparable effects on this surfactant. These findings support the conclusion that the primary effect is the presence of electrolyte rather than the specific identity of the salt, although sulfate exhibited the strongest cloud-point depression for the shorter-chain surfactants, consistent with its stronger salting-out effect predicted by the Hofmeister series.

## Experimental

### Reagents

Epoxidised linseed oil (Laroflex), toluene (Sigma-Aldrich, 99.5%), poly(ethylene glycol) (PEG) average *M*_n_ 400 Da, ∼950–1050 Da and average *M*_w_ 1500 Da (Sigma-Aldrich), and monomethyl poly(ethylene glycol) (MePEG) average *M*_n_ 400 and 750 Da (Alfa Aesar), sodium sulfate anhydrous (Avishkar, 99%), sodium chloride (BDH, 99.5%) and sodium nitrite (BDH, 99.5%). Magnesium sulphate anhydrous (Fisher, 99%), dichloromethane (Fisher, 99%), CDCl_3_ (Sigma-Aldrich, 99.8 atom), ultra-pure 18.2 mΩ Millipore water and distilled water.

### Instrumentation

NMR spectra were obtained on a Jeol 400 NMR spectrometer using CDCl_3_ solvent. IR spectra were obtained by running crude or purified samples neat on a Bruker Vertex 70 fitted with Specac Golden Gate ATR. SEC system was equipped with an Agilent 1260 Infinity or 1200 series HPLC pump, paired with variable wavelength UV detectors (1290 Infinity II) and dual refractive index/viscosity detectors. SEC was performed using tetrahydrofuran (≥99.9%, Sigma-Aldrich) as the eluent at a flow rate of 1 mL min^−1^ at 60 °C. Separation was achieved using a guard column (8 × 50 mm) connected in series to three GRAM columns (8 × 300 mm; nominal pore sizes 30, 100 and 1000 Å; Polymer Standards Service, Germany). The system was calibrated using narrowly dispersed poly(methyl methacrylate) (PMMA) standards (Polymer Standards Service), and the number-average molecular weight (*M*_n_), weight-average molecular weight (*M*_w_) and dispersity (*Đ*) of the surfactants were determined relative to the calibration curve. Dynamics studies and surface tension and critical micelle concentration (CMC) measurements on synthesised surfactants were recorded on Sinterface maximum bubble pressure tensiometer and Kruss DSA 100 tensiometer coupled with a dosing unit respectively.

### Preparation of catalyst

Iron montmorillonite catalyst (Fe-mont) was prepared by dissolving 1.62 g (10 mmol) iron(iii) chloride in 180 mL distilled water and thereafter adding 7 g neutral montmorillonite (Fulmont) as previously described.^9^

### Synthesis of surfactants from epoxidised linseed oil

PEG or MePEG (5 mmol) was placed in a 50 mL round-bottom flask and heated to 80 °C. Fe-mont catalyst (5 wt% relative to PEG or MePEG) was added, and the mixture was stirred for 2 minutes. Epoxidised linseed oil (0.817 mmol) was then added dropwise over 5–10 minutes *via* a dropping funnel. After the addition was complete, the reaction temperature was raised to 100 °C, and the mixture was stirred vigorously with the vessel covered. Reaction progress was monitored by ^1^H NMR spectroscopy using aliquots withdrawn at appropriate intervals. After 50 minutes, the reaction was stopped, and the mixture was transferred to a 250 mL beaker. Dichloromethane (50 mL) was added, and the mixture was filtered under reduced pressure to recover the catalyst. The filtrate was transferred to a separating funnel and washed with distilled water (25 mL), followed by brine. The organic layer was collected and dried over anhydrous MgSO_4_. The solvent was removed under reduced pressure using a rotary evaporator to afford the product. The product was characterised by IR spectroscopy, size exclusion chromatography (SEC), and ^1^H and proton-decoupled ^13^C NMR spectroscopy (SI).

### Dynamic surface tension, surface tension and critical micelle concentration determination (CMC)

Dynamic surface tension was determined following a reported procedure.^9^ A 1 g per L surfactant solution was prepared in a 10 mmol per L NaCl solution and aged for 24 h. Dynamic surface tension was measured using a maximum bubble pressure tensiometer with a capillary immersed 10 mm below the liquid surface. Measurements were recorded over bubble lifetimes up to 100 s.

Surface tension and critical micelle concentration (CMC) were determined according to a previously reported method.^11^ A 1 mg per mL surfactant stock solution was prepared and subsequently diluted to obtain a series of concentrations. Surface tension was measured, and the average surface tension at each concentration was determined. The resulting data were analysed using Origin 2022 software to establish CMC.

Maximum surface excess (*Γ*_max_) was calculated using the Gibbs adsorption equation for non-ionic surfactants,
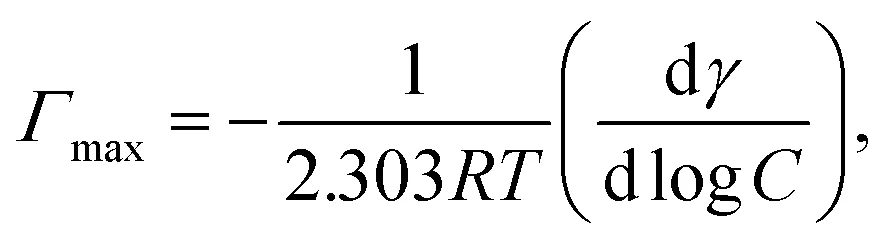
where the Gibbs prefactor *n* = 1 was adopted because the PEGylated triglycerides are electrically neutral and do not dissociate in aqueous solution. Although the products are polydisperse, each molecule behaves as a single non-ionic surfactant species in solution. The minimum molecular area was calculated from *A*_min_ = 1/(*N*_A_*Γ*_max_).

### Foam capacity and stability determination

Foam capacity and stability were determined according to a previously reported method.^28^ Aqueous surfactant solutions (50 mL, 0.25% w/v) were transferred into 100 mL graduated cylinders. Foam was generated at room temperature using a homogeniser operated at a fixed stirring speed for 1 minute. The foam capacity was evaluated immediately after homogenization by recording the foam volume.

Foam stability was assessed by monitoring the decrease in foam volume over time. Measurements were taken at 30 minutes intervals following foam generation by recording the reduction in foam volume in the graduated cylinder.

### Determination of relative emulsion capacity and stability

2 mL of distilled water and 2 mL of toluene were added to the 100 mg surfactant in the bottle to make a solution. Thereafter, the solution was homogenised with an orbital shaker at 200 rpm for 15 minutes. Emulsion stability was monitored at 2 day intervals for several weeks by observing phase separation of the emulsified solution.^11^

### Determination of cloud point

5 mL of 1 wt% surfactant solution was measured into a 10 mL glass tube, sealed with aluminium foil and immersed in a 250 mL beaker containing water fitted with a thermometer. Cloud points with and without the presence of salt were determined following a reported method.^11^

### Statistical analysis

Cloud point measurements were performed in triplicate and reported as mean ± standard deviation. Statistical analyses were conducted in SPSS using one-way analysis of variance (ANOVA) followed by Tukey's honestly significant difference (HSD) post hoc test to compare cloud points among salt treatments for each surfactant. Differences were considered statistically significant at *p* < 0.05.

## Conclusions

A series of novel bio-based non-ionic surfactants was successfully synthesised from epoxidised linseed oil through PEG- and MePEG-mediated epoxide ring-opening reactions. Spectroscopic and chromatographic analyses confirmed successful functionalisation of the triglyceride backbone, yielding a family of renewable PEGylated surfactants with tunable hydrophilic–lipophilic characteristics.

The surfactants exhibited excellent interfacial activity, efficient micellisation and favourable foaming, emulsifying and solution properties. Surface adsorption studies based on the Gibbs equation further demonstrated that PEG chain length strongly influenced molecular packing at the air–water interface. Surfactants bearing shorter PEG chains exhibited higher surface excess and smaller minimum molecular areas, enabling closer interfacial packing and greater foam persistence, whereas increasing PEG chain length increased the effective head-group area and promoted steric stabilisation, resulting in enhanced emulsion stability and improved hydrophilicity. Their cloud point behaviour and predictable response to electrolyte addition further confirm their suitability for formulation applications.

These findings demonstrate that the balance between the renewable hydrophobic linseed oil core and the PEG-derived hydrophilic segments can be systematically tailored to achieve desirable interfacial properties. The present work establishes epoxidised linseed oil as a versatile and sustainable platform for the development of high-performance non-ionic surfactants and highlights the potential of these materials as renewable alternatives to conventional petroleum-derived surfactants for industrial, household and personal care applications. Moreover, the synthetic strategy provides a flexible route for designing next-generation bio-based surfactants with application-specific performance.

## Author contributions

Joseph Ogunjobi: conceptualisation, data curation, formal analysis, investigation, resources, supervision, writing – original draft, review and editing. Osaretin Omoruyi: investigation, analysis, resources and writing – original draft.

## Conflicts of interest

Authors declare no conflict of interest.

## Supplementary Material

RA-016-D6RA05443A-s001

## Data Availability

Raw data on interfacial properties used in this study are available at Zenodo (https://doi.org/10.5281/zenodo.21719661). Supplementary information (SI): product characterisation, foam and emulsion properties. See DOI: https://doi.org/10.1039/d6ra05443a.
