# Supplementary material for: Sustainable non-ionic surfactants from epoxidised linseed oil: synthesis, interfacial properties, foaming behaviour, and emulsification performance
Source: RSC Adv. 2026 Aug 26;16(42):50738–49. doi: 10.1039/d6ra05443a (PMC13512901; doi:10.1039/d6ra05443a)
Supplement: Supplementary file 1 [file RA-016-D6RA05443A-s001.pdf]

Supplementary Information (SI) for RSC Advances.
This journal is © The Royal Society of Chemistry 2026

Sustainable Non-ionic Surfactants from Epoxidised Linseed Oil: Synthesis, Interfacial Properties, Foaming Behaviour, and Emulsification Performance

Joseph K. Ogunjobi[a, b*] and Osaretin E. Omoruyi[a]

[a] Department of Chemistry, The Federal University of Technology, PMB 704, Akure, Nigeria.

[b] DWI-Leibniz Institute for Interactive Materials, RWTH Aachen University, Forckenbeckstr. 50, D-5074, Aachen, Germany.

*Corresponding Author
Joseph Ogunjobi: josephogunjobi@outlook.com; jkogunjobi@futa.edu.ng
ORCID: 0000-0001-9276-7310

ELECTRONIC SUPPLEMENTARY INFORMATION

Table of Content

1.0 Proton and Carbon-13 NMR and IR data of synthesised surfactants……………S2-S3

2.0 Size Exclusion Chromatography…………………………………………………S3-S4

3.0 Foam Capacity and Stability…………………………………………………………S5

4.0 Extraction of Maximum Surface Excess and Minimum Molecular Area…………S5-S6

5.0 Relative Emulsion Capacity and Stability……………………………………………S7

1.0 Proton and Carbon-13 NMR and IR data of synthesised surfactants

ELO400, ^{1}H NMR (700 MHz, $CDCl_3$, ∂ ppm): 0.85 (~6H, 2x $C\mathit{H}_3CH_2$-), 0.96 (3H,$C\mathit{H}_3CH_2CH(OH)$-), 1.26 (56H, -$(C\mathit{H}_2)_{34}$-), 2.29 (6H, t, 3x -$C\mathit{H}_2COO$-), 3.07 (~1H, m, -$C\mathit{H}((OCH_2CH_2)_9OH)$-), 3.64 (226H, m, 6x -$\mathit{H}C(OH)CH((OC\mathit{H}_2C\mathit{H}_2)_9OH)$-), 4.12 (2H, m, -$COOCH(CH_2OOC\text{-})C\mathit{H}_2OOC$-), 4.27 (2H, m, -$COOCH(C\mathit{H}_2OOC\text{-})CH_2OOC$-), 5.23 (1H, quint, -$COOC\mathit{H}(CH_2OOC\text{-})CH_2OOC$-); ^{13}C NMR (175 MHz, $CDCl_3$, ∂ ppm): 14.11, 22.66, 24.82, 25.62, 29.08, 29.24, 29.32, 29.44, 29.58, 29.62, 29.66, 29.88, 31.11, 31.84, 31.86, 31.88, 33.04, 34.01, 61.52, 61.59, 61.63, 62.05, 63.01, 63.34, 68.82, 69.16, 69.96, 70.13, 70.19, 70.24, 70.27, 70.40, 70.43, 70.49-70.57, 70.78, 72.36, 72.54, 72.56, 72.63, 72.88, 73.40, 78.53, 84.55, 172.82, 173.21; IR (ATR, ν, cm^{-1}), 3454 (O-H str.), 2922 (s. CH_3 str.), 2867 (s. CH_2 str.), 1739 (C=O str.), 1460 (a. CH_3 bend), 1350 (s. CH_2 bend), 1296 (end group C-O- str.), 1249 (C-O- str.), 1102 (a. C-O-C str.). 2.33 g total product mass recovered, equivalent to 82.9% isolated yield.

ELO400M, ^{1}H NMR (700 MHz, $CDCl_3$, ∂ ppm): 0.91 (~8H, 2x $C\mathit{H}_3CH_2$-, $C\mathit{H}_3CH_2CH(OH)$-), 1.25 (54H, -$(C\mathit{H}_2)_{34}$-), 2.28 (6H, t, 3x $C\mathit{H}_2COO$-), 3.06 (~1H, m, $C\mathit{H}((OCH_2CH_2)_9OCH_3)$-), 3.35 (21H, s, 6x $CH((OCH_2CH_2)_9OC\mathit{H}_3)$-), 3.61 (222H, m, 6x -$\mathit{H}C(OH)CH((OC\mathit{H}_2C\mathit{H}_2)_9OCH_3)$-), 4.12 (2H, m, -$COOCH(CH_2OOC\text{-})C\mathit{H}_2OOC$-), 4.26 (2H, m, -$COOCH(C\mathit{H}_2OOC\text{-})CH_2OOC$-), 5.23 (1H, quint, -$COOC\mathit{H}(CH_2OOC\text{-})CH_2OOC$-); ^{13}C NMR (175 MHz, $CDCl_3$, ∂ ppm): 14.11, 22.65, 24.82, 29.07, 29.23, 29.32, 29.66, 31.10, 31.88, 33.95, 58.99, 61.63, 63.00, 68.82, 69,76, 70.24-70.56, 71.88, 72.55, 84.58; IR (ATR, ν, cm^{-1}), 3446 (O-H str.), 2868 (s. CH_2 str.), 1741 (C=O str.), 1457 (a. CH_3 bend), 1350 (s. CH_2 bend), 1297 (end group C-O- str.), 1248 (C-O- str.), 1104 (a. C-O-C str.). 2.27 g total product mass recovered, equivalent to 80.7% isolated yield.

ELO750M, ^{1}H NMR (700 MHz, $CDCl_3$, ∂ ppm): 0.89 (~7H, 2x $C\mathit{H}_3CH_2$-, $C\mathit{H}_3CH_2CH(OH)$-), 1.42 (52H, -$(C\mathit{H}_2)_{34}$-), 2.28 (6H, t, 3x -$C\mathit{H}_2COO$-), 3.07 (~1H, m, -$C\mathit{H}((OCH_2CH_2)_{16}OCH_3)$-), 3.35 (19H, s, 6x -$CH((OCH_2CH_2)_{16}OC\mathit{H}_3)$-), 3.55 (376H, m, 6x -$\mathit{H}C(OH)CH((OC\mathit{H}_2C\mathit{H}_2)_{16}OCH_3)$-), 4.11 (2H, m, -$COOCH(CH_2OOC\text{-})C\mathit{H}_2OOC$-), 4.27 (2H, m, -$COOCH(C\mathit{H}_2OOC\text{-})CH_2OOC$-), 5.22 (1H, quint, -$COOC\mathit{H}(CH_2OOC\text{-})CH_2OOC$-); ^{13}C NMR (175 MHz, $CDCl_3$, ∂ ppm): 14.11, 22.65, 24.81, 29.07-29.32, 29.61, 29.65, 31.88, 34.00, 58.99, 61.63, 62.03, 62.99, 68.81, 69.77, 70.28-70.55, 71.88, 72.48; IR (ATR, ν, cm^{-1}), 3481 (O-H str.), 2870 (s. CH_2 str.), 1740 (C=O str.), 1465 (a. CH_3 bend), 1345 (s. CH_2 bend), 1281 (end group C-O- str.), 1243 (C-O- str.), 1110 (a. C-O-C str.). 4.15 g total product mass recovered, equivalent to 94.4% isolated yield.

ELO1050, ^{1}H NMR (700 MHz, $CDCl_3$, ∂ ppm): 0.92 (~8H, 2x $C\mathit{H}_3CH_2$-, $C\mathit{H}_3CH_2CH(OH)$-), 1.43 (57H, -$(C\mathit{H}_2)_{34}$-), 2.30 (6H, t, 3x -$C\mathit{H}_2COO$-), 3.08 (~1H, m, -$C\mathit{H}((OCH_2CH_2)_{22}OH)$-), 3.63 (593H, m, 6x -$\mathit{H}C(OH)CH((OC\mathit{H}_2C\mathit{H}_2)_{22}OH)$-), 4.13 (2H, m, -$COOCH(CH_2OOC\text{-})C\mathit{H}_2OOC$-), 4.28 (2H, m, -$COOCH(C\mathit{H}_2OOC\text{-})CH_2OOC$-), 5.24 (1H, quint, -$COOC\mathit{H}(CH_2OOC\text{-})CH_2OOC$-); ^{13}C NMR (175 MHz, $CDCl_3$, ∂ ppm): 14.13, 22.67, 24.83, 29.24-29.67, 31.89, 34.14, 61.66, 63.00, 68.83, 69.17, 70.30-70.58, 70.78, 72.49; IR (ATR, ν, cm^{-1}), 3451 (O-H str.), 2886 (s. CH_2 str.), 1739 (C=O str.), 1467 (a. CH_3 bend), 1360, 1343 (s. CH_2 bend), 1280 (end group C-O- str.), 1241 (C-O- str.), 1108 (a. C-O-C str.). 5.32 g total product mass recovered, equivalent to 94.8% isolated yield.

ELO1500, ^{1}H NMR (700 MHz, $CDCl_3$, ∂ ppm): 0.90 (~8H, 2x $C\mathit{H}_3CH_2$-, $C\mathit{H}_3CH_2CH(OH)$-), 1.42 (56H, -$(C\mathit{H}_2)_{34}$-), 2.30 (6H, t, 3x -$C\mathit{H}_2COO$-), 3.07 (~1H, m, -$C\mathit{H}((OCH_2CH_2)_{34}OH)$-), 3.60 (783H, m, 6x -$\mathit{H}C(OH)CH((OC\mathit{H}_2C\mathit{H}_2)_{34}OH)$-), 4.12 (2H, m, -$COOCH(CH_2OOC\text{-})C\mathit{H}_2OOC$-), 4.29 (2H, m, -$COOCH(C\mathit{H}_2OOC\text{-})CH_2OOC$-), 5.23 (1H, quint, -$COOC\mathit{H}(CH_2OOC\text{-})CH_2OOC$-); ^{13}C NMR (175 MHz, $CDCl_3$, ∂ ppm): 14.13, 22.63, 24.79, 29.25, 29.66, 31.89, 34.13, 61.66, 69.77, 70.30-70.58,

72.50; IR (ATR, ν, cm[-1]), 3442 (O-H str.), 2886 (s. CH_2 str.), 1741 (C=O str.), 1467 (a. CH_3 bend), 1360, 1342 (s. CH_2 bend), 1280 (end group C-O- str.), 1241 (C-O- str.), 1107 (a. C-O-C str.). 6.71 g total product mass recovered, equivalent to 82.1% isolated yield.

2.0 Size Exclusion Chromatography

The SEC (size exclusion chromatography) chromatograms provide valuable information on the molecular weight distribution of the PEGylated epoxidised linseed oil (ELO) surfactants. The SEC chromatograms show broad molecular weight distributions for all four surfactants, which is expected for products synthesised through the ring-opening of epoxidised triglycerides with polymeric PEGs. Unlike small-molecule surfactants, these products comprise mixtures of molecules differing in both the degree of epoxidation of the parent oil and the extent of PEG substitution. Consequently, the products are intrinsically polydisperse rather than discrete compounds.

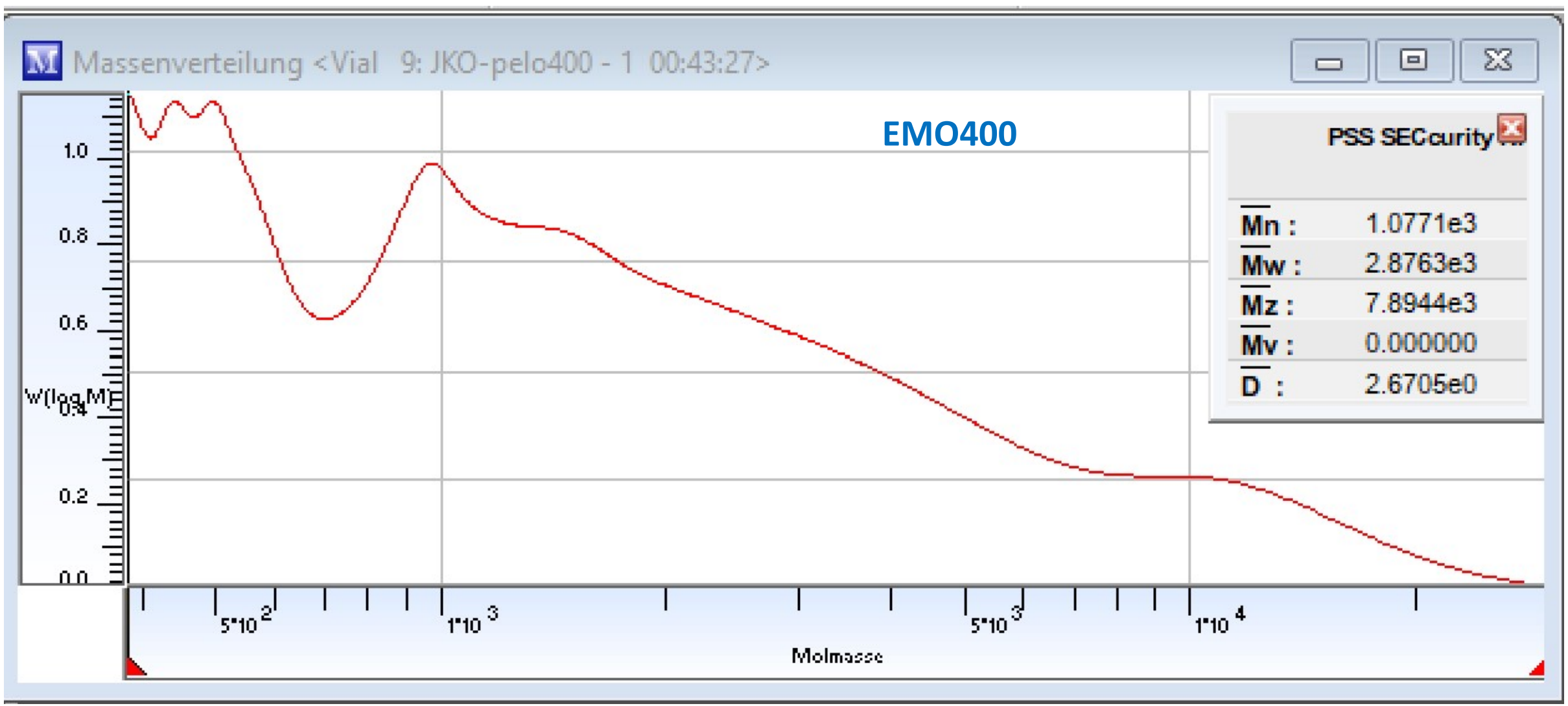

Figure S 1 SEC chromatogram of ELO400 surfactant

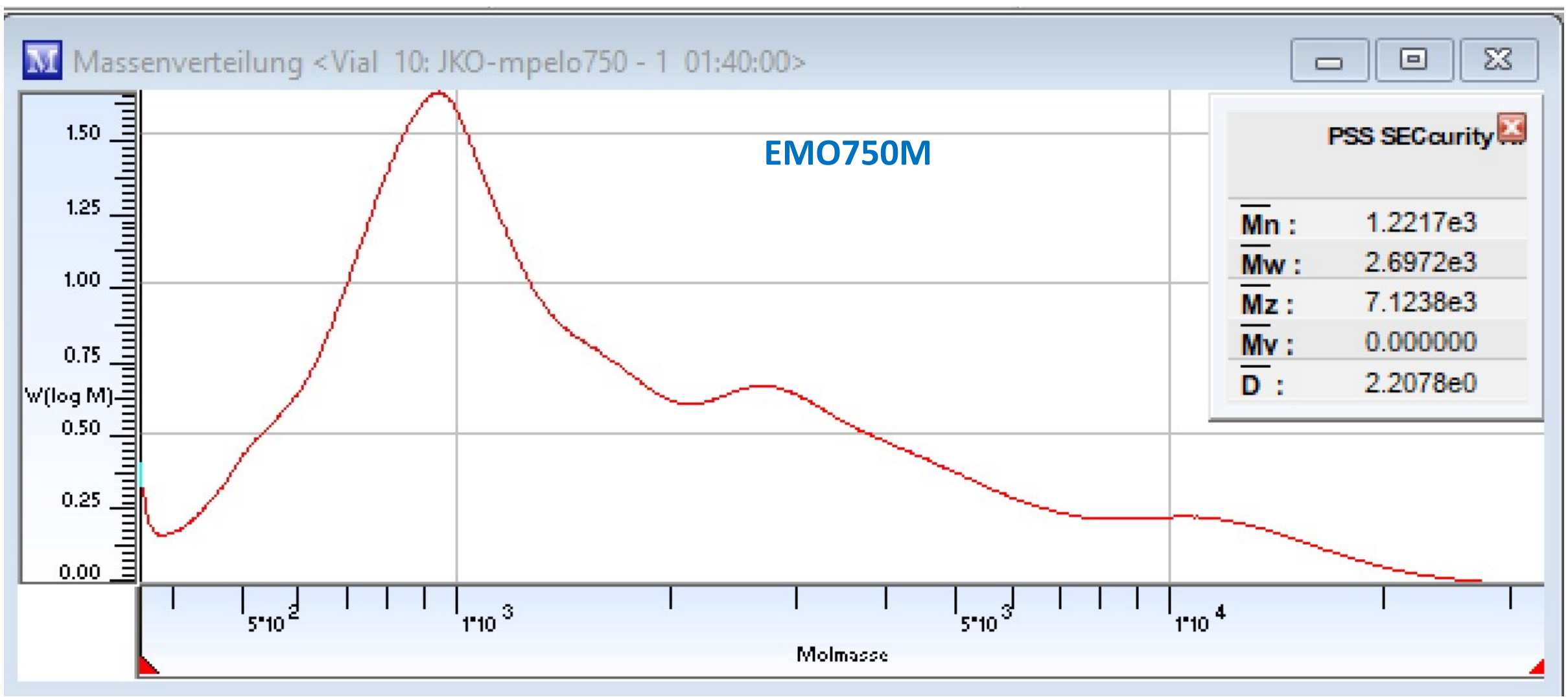

Figure S 2 SEC chromatogram of ELO750M surfactant

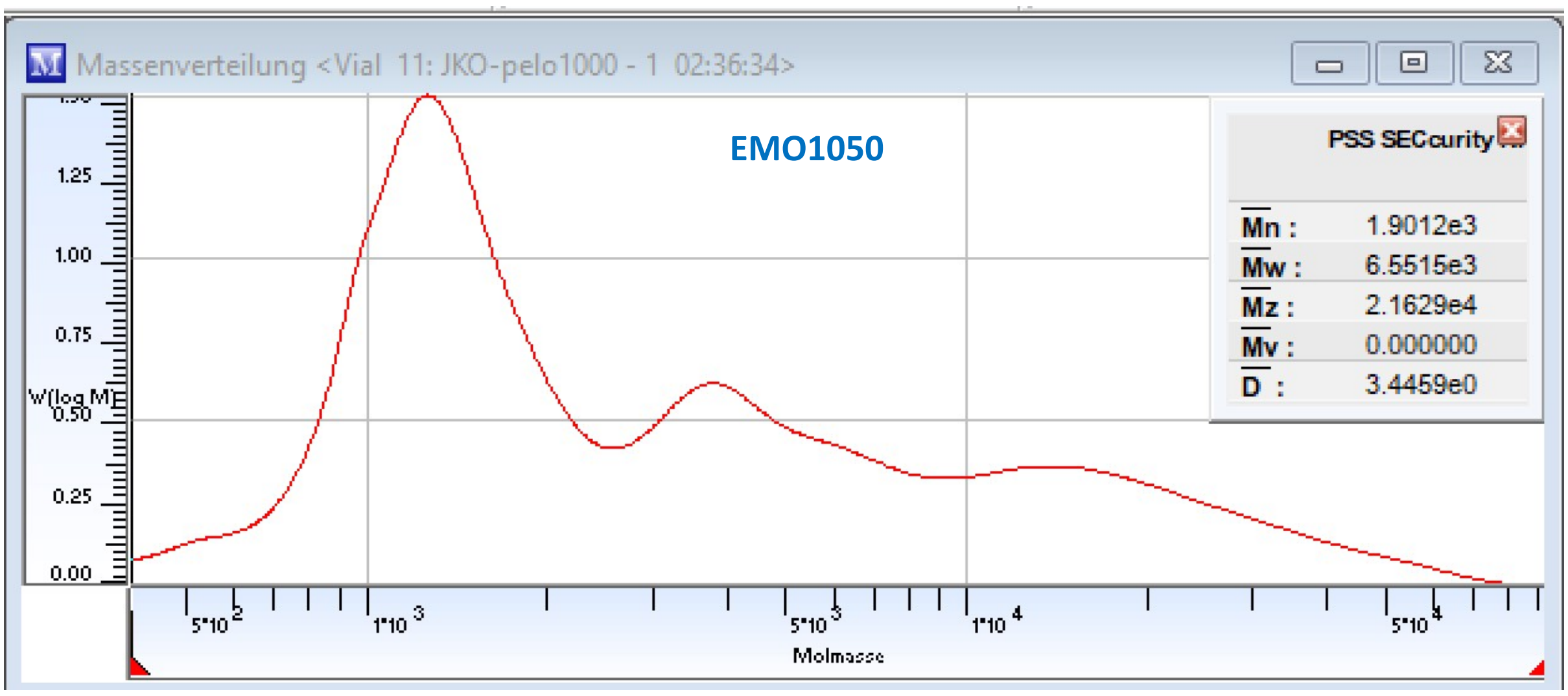

Figure S 3 SEC chromatogram of ELO1050 surfactant

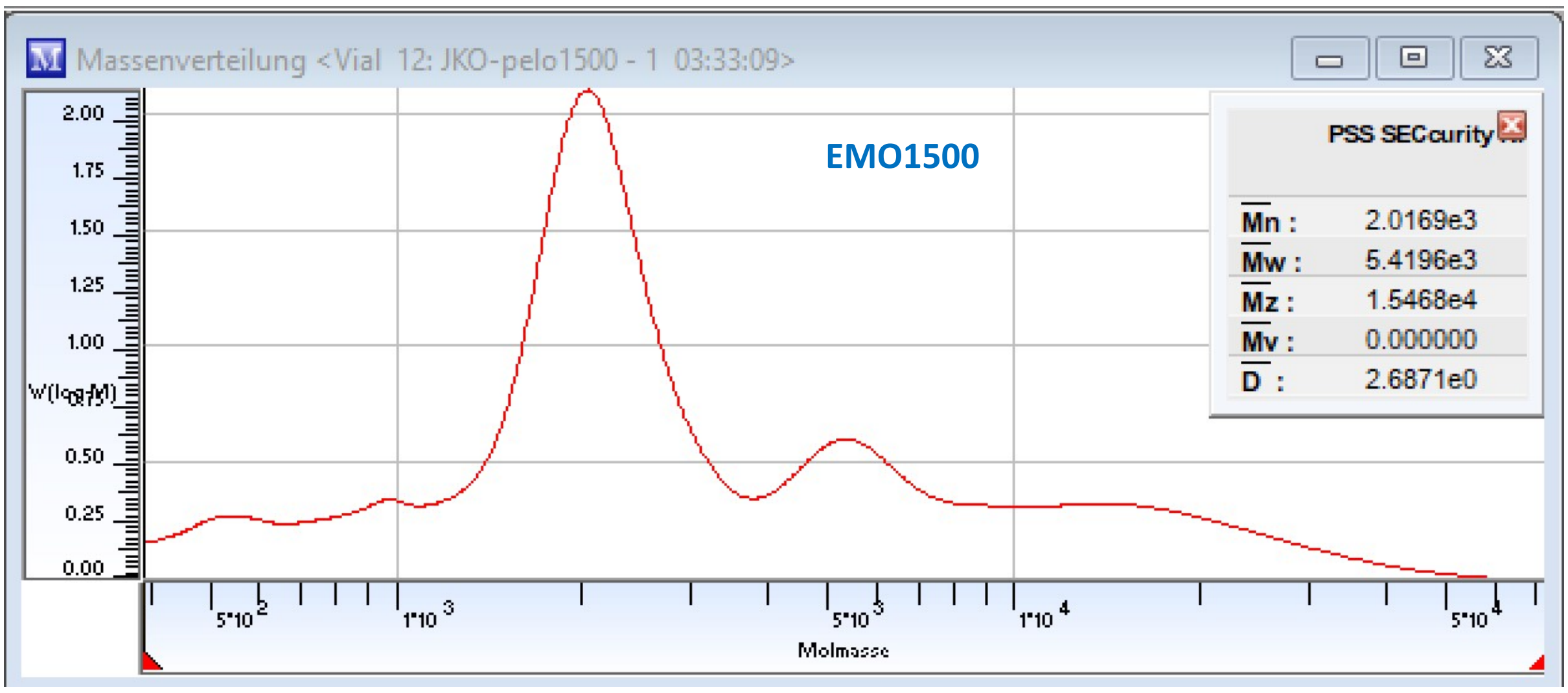

Figure S 4 SEC chromatogram of ELO1500 surfactant

Table S1 SEC (size exclusion chromatography) chromatograms provide valuable information on the molecular weight distribution of the PEGylated epoxidised linseed oil (ELO) surfactants. Theoretical M_n are derived from ethoxylate number assuming that all six epoxide groups on epoxidised linseed oil (ELO) are opened and substituted by PEG chain.

**Surfactant**	**M_n (g mol⁻¹)**	**M_w (g mol⁻¹)**	**M_z (g mol⁻¹)**	**Đ (Mw/Mn)**	**Theoretical M_n (Da)**	**SEC/Theoretical (%)**
ELO400	1077	2876	7894	2.67	3440	31.3
ELO750M	1222	2697	7124	2.21	5380	22.7
ELO1050	1901	6552	21629	3.45	6870	27.7
ELO1500	2017	5420	15468	2.69	10000	20.2

3.0 Foam Capacity and Stability

Table S2 Foam volume recorded at certain time after foam generation to as a measure of stability of Epoxidised Linseed Oil-Based Surfactants from ELO400, ELO1050, ELO1500, ELO400M and ELO750M. (0.25% surfactant concentration).

Time (Min)	Foam Volume (mL) ELO400	Foam Volume (mL) ELO1050	Foam Volume (mL) ELO1500	Foam Volume (mL) ELO400M	Foam Volume (mL) ELO750M
0	25	36	32	12	15
30	17	20	16	7	9
60	15	14	13	7	8
90	15	10	11	6	7
120	15	8	8	6	6

4.0 Extraction of Maximum Surface Excess and Minimum Molecular Area

Using the surface tension data, the Gibbs adsorption parameters were estimated from the linear pre-CMC region of the surface tension versus log concentration plots.

For a non-ionic surfactant, the Gibbs adsorption equation is

$$\Gamma_{max} = -\frac{1}{2.303RT}\left(\frac{d\gamma}{dLogC}\right)$$

where

- Γmax = maximum surface excess (mol m^{-2})
- R=8.314 J $mol^{-1}K^{-1}$
- T=298 K
- γ = surface tension (N m^{-1})
- C = surfactant concentration.

The minimum molecular area is then

$A_{min}=1/(N_A\Gamma_{max})$

where N_A is Avogadro's constant.

Choice of Gibbs prefactor

For these surfactants, n = 1 was used because

- the surfactants are non-ionic;

- no counterions are produced upon adsorption;
- the PEGylated triglycerides remain electrically neutral in solution.

Although the products are polydisperse and possess multiple PEG chains, the Gibbs equation depends on the number of independently diffusing species, not on the number of hydrophilic chains attached to one molecule. Each PEGylated triglyceride therefore behaves as one non-ionic surfactant molecule, making the classical non-ionic Gibbs equation appropriate.

This approach is widely adopted for ethoxylated non-ionic surfactants and polymeric PEG surfactants (Rosen & Kunjappu, *Surfactants and Interfacial Phenomena*, 4th ed.; Holmberg et al., *Surfactants and Polymers in Aqueous Solution*).

Estimated slopes

Using the approximately linear adsorption region immediately before the CMC gives slopes as presented in Table S3.

Table S3 Slope of ***dγ/dlog C*** *curve plot immediately before CMC was reached.*

Surfactant	Slope (dγ/dlog C) ($mN\ m^{-1}\ decade^{-1}$)
ELO400	−29.8
ELO750M	−25.6
ELO1050	−22.0
ELO1500	−23.6

Table S4 Calculated Gibbs adsorption parameters

Surfactant	Γmax ($\times 10^{-6}$ mol m^{-2})	Amin (nm^2 $molecule^{-1}$)
ELO400	4.93	0.34
ELO750M	4.23	0.39
ELO1050	3.64	0.46
ELO1500	3.90	0.43

5.0 Relative Emulsion Capacity and Stability

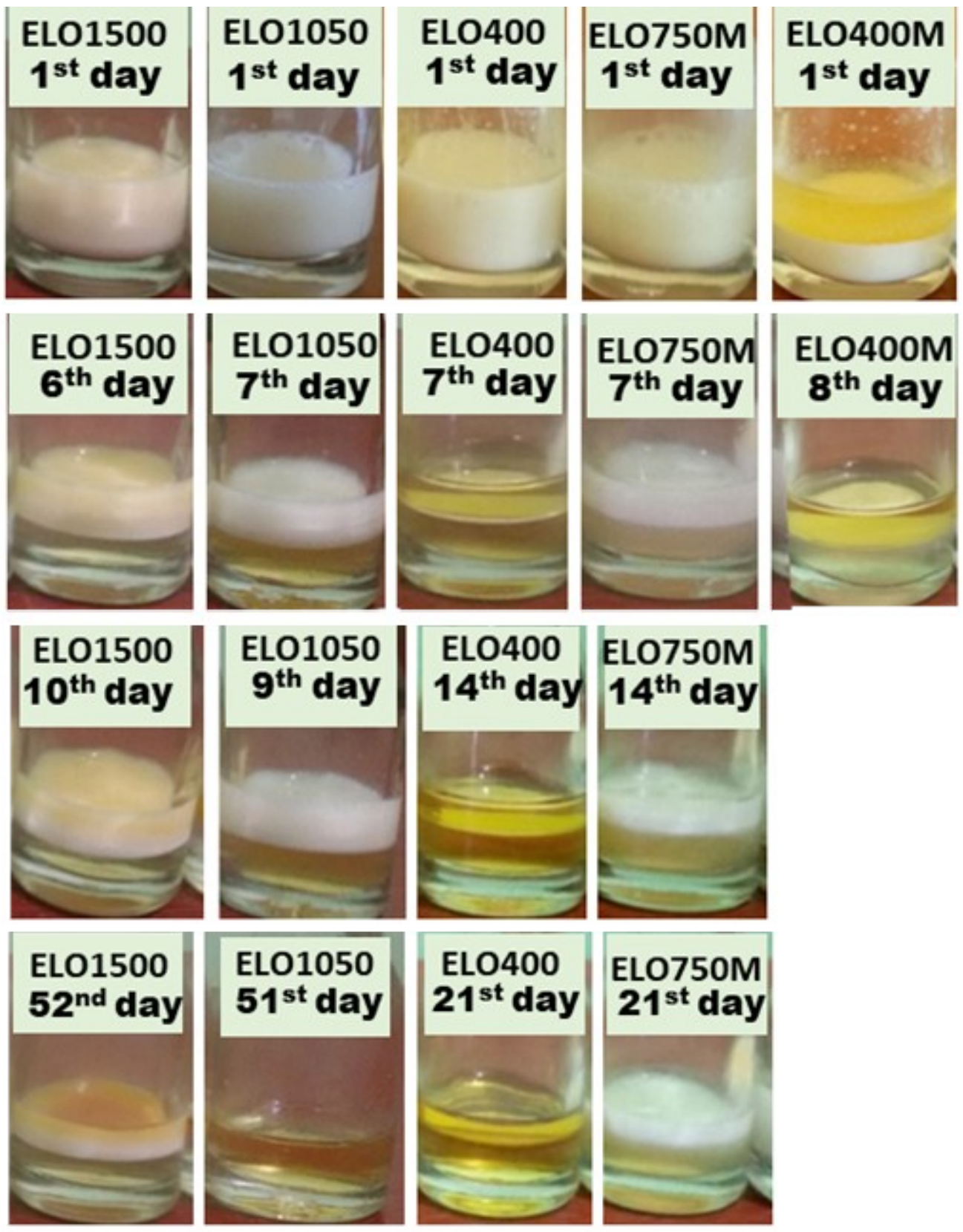

Figure S 5 Batch emulsion formation of ELO-based surfactants and their stability were observed over several days.
